# Deconvolution of DNA methylation identifies differentially methylated gene regions on 1p36 across breast cancer subtypes

**DOI:** 10.1038/s41598-017-10199-z

**Published:** 2017-09-14

**Authors:** Alexander J. Titus, Gregory P. Way, Kevin C. Johnson, Brock C. Christensen

**Affiliations:** 1 Program in Quantitative Biomedical Sciences, Geisel School of Medicine at Dartmouth, Hanover, NH 03755 USA; 2 Genomics and Computational Biology Graduate Program, University of Pennsylvania, Philadelphia, PA 19104 USA; 3 The Jackson Laboratory for Genomic Medicine, Farmington, CT 06032 USA; 4 Department of Epidemiology, Geisel School of Medicine at Dartmouth, Hanover, NH 03755 USA; 5 Department of Molecular and Systems Biology, Geisel School of Medicine at Dartmouth, Hanover, NH 03755 USA; 6 Department of Community and Family Medicine, Geisel School of Medicine at Dartmouth, Hanover, NH 03755 USA

## Abstract

Breast cancer is a complex disease consisting of four distinct molecular subtypes. DNA methylation-based (DNAm) studies in tumors are complicated further by disease heterogeneity. In the present study, we compared DNAm in breast tumors with normal-adjacent breast samples from The Cancer Genome Atlas (TCGA). We constructed models stratified by tumor stage and PAM50 molecular subtype and performed cell-type reference-free deconvolution to control for cellular heterogeneity. We identified nineteen differentially methylated gene regions (DMGRs) in early stage tumors across eleven genes (*AGRN, C1orf170, FAM41C, FLJ39609, HES4, ISG15, KLHL17, NOC2L, PLEKHN1, SAMD11, WASH5P*). These regions were consistently differentially methylated in every subtype and all implicated genes are localized to the chromosomal cytoband 1p36.3. Seventeen of these DMGRs were independently validated in a similar analysis of an external data set. The identification and validation of shared DNAm alterations across tumor subtypes in early stage tumors advances our understanding of common biology underlying breast carcinogenesis and may contribute to biomarker development. We also discuss evidence of the specific importance and potential function of 1p36 in cancer.

## Introduction

Invasive breast cancer is a complex disease characterized by diverse etiologic factors^1^. Key genetic and epigenetic alterations are recognized to drive tumorigenesis and serve as gate-keeping events for disease progression^2^. Early DNA methylation (DNAm) events have been shown to contribute to breast cancer development^3^. Importantly, DNAm alterations have been implicated in the transition from normal tissue to neoplasia^4, 5^ and from neoplasia to metastasis^6^. Furthermore, patterns of DNAm are known to differ across molecular subtypes of breast cancer^7^ – Luminal A (LumA), Luminal B (LumB), Her2-enriched and Basal-like – identified based on the prediction analysis of microarray 50 (PAM50) classification^8^. However, while DNAm differences across breast cancer subtypes have been explored, similarities across subtypes are less clear^9^. Such similarities found in early stage tumors can inform shared biology underpinning breast carcinogenesis and – as similarities would be agnostic to subtype – potentially contribute to biomarkers for early detection.

Studying DNAm in bulk tumors is complicated by disease heterogeneity. Heterogeneity is driven by many aspects of cancer biology including variable cell-type proportions found in the substrate used for molecular profiling^10^. Different proportions of stromal, tumor, and infiltrating immune cells may confound molecular profile classification when comparing samples^11^ because cell types have distinct DNAm patterns^12–14^. The potential for cell–type confounding prompted the development of statistical methods to adjust for variation in cell-type proportions in blood^15^ and solid tissue^16, 17^. One such method, *RefFreeEWAS*, is a reference-free deconvolution method and does not require a reference population of cells with known methylation patterns and is agnostic to genomic location when performing deconvolution^18^. Instead, the unsupervised method infers underlying cell-specific methylation profiles through constrained non-negative matrix factorization (NMF) to separate cell-specific methylation differences from actual aberrant methylation profiles observed in disease states. This method has previously been shown to effectively determine the cell of origin in breast tumor phenotypes^19^.

We applied *RefFreeEWAS* to The Cancer Genome Atlas (TCGA) breast cancer DNAm data and estimated cell proportions across the set. We compared tumor DNAm with adjacent normal tissue stratified by tumor subtype^9^ and identified common early methylation alterations across molecular subtypes that are independent of cell type composition. We identified a specific chromosomal location, 1p36.3, that harbors all 19 of the differentially methylated regions that are in common to early stage breast cancer subtypes. 1p36 is a well-studied and important region in many different cancer types, but there remain questions about how it may impact carcinogenesis and disease progression^20^. Our study provides evidence that methylation in this region may provide important clues about early events in breast cancer. We also performed *RefFreeEWAS* on an independent validation set (GSE61805) and confirmed these results^21^.

## Results

### DNA methylation deconvolution

Subject age and tumor characteristic data, stratified by PAM50 subtype and stage, is provided in Table 1 for the 523 TCGA tumors analyzed. DNAm data was collected using the Illumina HumanMethylation450 (450 K) array. TCGA breast tumor sample purity, estimated by pathologists from histological slides, was consistent across PAM50 subtypes and stages indicating that the conclusions of our analyses are not predominantly a result of large differences in tumor purity (Supplementary Fig. S1). To correct for cell-proportion differences across tumor samples, we estimated the number of cellular methylation profiles contributing to the mixture differences by applying NMF to the matrix of beta values, which resulted in individual sample specific dimensionality estimates indicating diverse cellular methylation profiles (Supplementary Table S1). The reference-free deconvolution altered the number of significant differentially methylated CpGs and the magnitude of their methylation values across all models that compared breast tumor methylation with adjacent normal samples (Supplementary Fig. S2).

**Table 1:** Sample information stratified by PAM50 subtype.

	Basal-like	Her2	Luminal A	Luminal B	Total with Assignment	Normal-adjacent	Validation
TCGA tumors	86	31	279	127	523	124	186
Age, mean (SD)	56.8 (12.8)	60 (12.8)	58 (13.5)	57.1 (12.6)	57.8 (13.1)	57.6 (12.7)	Unknown
Stage*, n (%)	—	—	—	—	—	—	—
Early (I/II)	70 (81%)	20 (65%)	207 (74%)	84 (66%)	381 (73%)	NA	Unknown
Late (III/IV)	14 (16%)	10 (32%)	69 (25%)	42 (33%)	135 (26%)	NA	Unknown
Missing	2 (2%)	1 (3%)	3 (1%)	1 (1%)	7 (1%)	NA	Unknown

*AJCC characterized stage, provided by TCGA.

### Subtype specific methylation patterns

**Figure 1:**
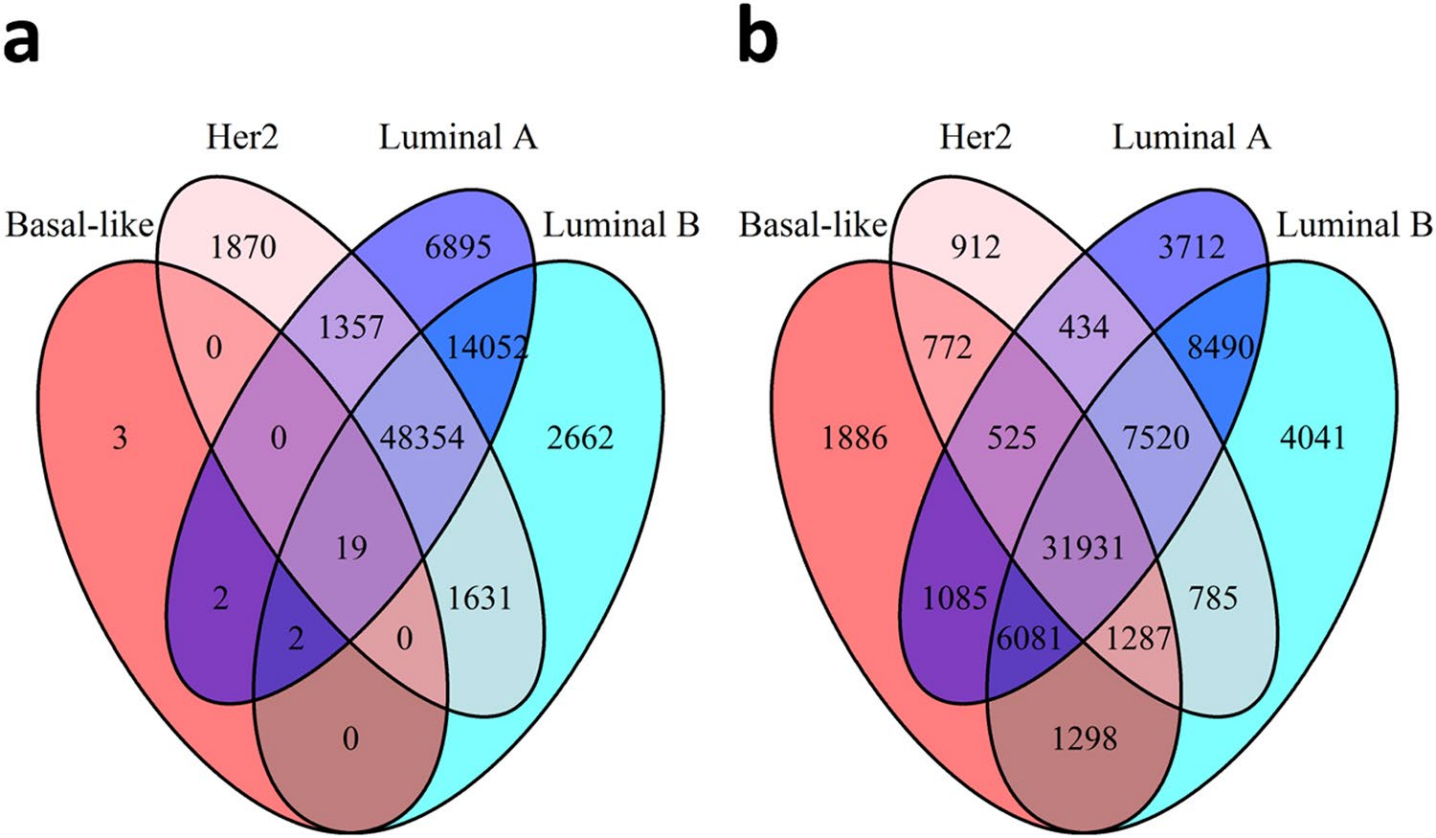
Numbers of overlapping differentially methylated gene regions in (**a**) early stage tumors (DMGR total = 76,847) and (**b**) late stage tumors (DMGR total = 70,759) stratified by Basal-like, Her2, Luminal A, and Luminal B PAM50 subtypes with a Q-value cutoff of 0.01.

In early stage tumors (AJCC stage I/II, n = 381), we identified a set of nineteen differentially methylated gene regions (DMGRs) shared among Luminal A, Luminal B, Her2, and Basal-like subtypes (DMGRs *Q* < 0.01, Fig. 1a). In the late stage tumors (AJCC stage III/IV, n = 135), we identified 31,931 DMGRs in common across subtypes (Fig. 1b). DMGRs are identified independent of both subtype and hypo/hypermethylation status and serve to prioritize specific regions of interest in breast carcinogenesis for follow up.

Subtype specific methylation patterns in early stage tumors were most divergent for Basal-like tumors versus other types, while in late stage tumors methylation alterations in Luminal B tumors were most divergent (Supplementary Table S2).

To test if collapsing CpGs by genomic region biased the detection of differential methylation, we also performed RefFreeEWAS using regions defined by CpG density and genomic distance (i.e. CpG island, Shore, Shelf, Open Sea) as indicated in the annotation file from Illumina. Defining regions by CpG island context indicated similar results (Supplementary Fig. S3), although we observed a lower number of common DMGRs. We used gene region probe collapsing for all downstream analyses. CpGs were assigned to gene regions based on the annotation file variable “*UCSC_RefGene_Group”* as detailed in the methods section.

We identified nineteen DMGRs with common methylation alterations among early stage tumor subtypes in comparison with normal tissues that were annotated to eleven genes via the 450 K annotation data set provided by Illumina: *AGRN, C1orf170, FAM41C, FLJ39609, HES4, ISG15, KLHL17, NOC2L, PLEKNH1, SAMD11*, and *WASH5P* (Table 2).

**Table 2:** Nineteen differentially methylated gene regions in common to early stage tumors.

*DMGR*	*Alternate* *Gene Name*	*Basal* *Med Q*	*Her2* *Med Q*	*Lum A* *Med Q*	*Lum B* *Med Q*	*Any late stage	*All late stage	*Present in* *validation*	*Validation* *Median Q*	Genomic position of assodciated gene
AGRN Body	AGNR	2.4E-06	1.7E-04	1.8E-07	1.3E-06	Y	—	Y	7.80E-21	chr1:1,020,123–1,056,116
C1orf170 Body	PERM1	4.0E-11	1.7E-05	5.5E-09	9.7E-04	Y	Y	Y	1.31E-08	chr1:975,205–981,029
C1orf170 TSS1500	PERM1	5.4E-04	6.5E-03	7.8E-06	6.8E-05	Y	—	Y	9.23E-03	chr1:975,205–981,029
FAM41C Body	FAM41C	4.1E-03	4.2E-08	1.2E-20	3.4E-03	Y	Y	Y	8.25E-10	chr1:868,071–876,903
FAM41C TSS1500	FAM41C	3.3E-04	1.1E-04	8.4E-05	1.0E-34	Y	Y	Y	1.75E-24	chr1:868,071–876,903
FLJ39609 TSS200	LOC100130417	1.3E-04	6.0E-05	2.9E-06	3.7E-04	Y	Y	Y	5.24E-06	chr1:916,865–921,016
HES4 TSS1500	HES4	3.1E-03	5.2E-04	7.8E-05	2.2E-04	Y	—	Y	5.06E-04	chr1:998,964–1,000,111
ISG15 Body	ISG15	3.1E-07	2.4E-04	1.2E-05	3.6E-04	Y	Y	—	1.03E-01	chr1:1,013,423–1,014,540
KLHL17 3′UTR	KLHL17	3.1E-05	5.5E-07	3.8E-16	2.3E-03	Y	Y	Y	3.99E-08	chr1:960,587–965,715
KLHL17 Body	KLHL17	5.9E-06	1.1E-04	7.9E-04	7.2E-05	Y	—	Y	1.60E-06	chr1:960,587–965,715
NOC2L Body	NOC2L	3.2E-04	6.2E-04	6.6E-05	2.4E-06	Y	Y	Y	4.90E-11	chr1:944,204–959,290
PLEKHN1 3′UTR	PLEKHN1	5.2E-16	4.7E-06	3.1E-07	7.7E-06	Y	—	Y	9.83E-09	chr1:966,497–975,108
PLEKHN1 Body	PLEKHN1	8.9E-10	2.7E-09	7.6E-29	1.7E-30	Y	Y	Y	5.87E-18	chr1:966,497–975,108
PLEKHN1 TSS1500	PLEKHN1	3.1E-05	5.5E-07	2.6E-06	3.6E-07	Y	Y	Y	3.99E-08	chr1:966,497–975,108
PLEKHN1 TSS200	PLEKHN1	1.6E-18	5.8E-10	1.4E-03	1.2E-03	Y	Y	Y	2.93E-10	chr1:966,497–975,108
SAMD11 5′UTR	SAMD11	3.6E-03	7.2E-12	1.0E-09	2.2E-08	Y	Y	Y	4.59E-11	chr1:925,738–944,575
SAMD11 Body	SAMD11	7.1E-08	2.5E-08	8.5E-06	2.0E-04	Y	Y	Y	3.26E-23	chr1:925,738–944,575
SAMD11 TSS1500	SAMD11	2.4E-03	6.1E-04	8.6E-04	1.0E-03	Y	Y	Y	2.02E-05	chr1:925,738–944,575
WASH5P Body	WASH7P	2.9E-03	9.8E-03	1.6E-03	1.3E-05	Y	—	—	7.01E-02	chr1:14,362–29,370

*Reference to any or all breast cancer subtypes in late stage tumors.

In the eleven genes identified, we observed differential methylation in regions including gene body, promoter (TSS1500, and TSS200), and 3′UTR. Across all four subtypes, we identified DMGRs with both hyper-methylation (*AGRN* – gene body; *FAM41C –* TSS1500; *KLHL17 –* 3′UTR & gene body; *PLEKHN1* – 3′UTR, gene body, & TSS1500; *SAMD11 –* 5′UTR, gene body, & TSS1500) and hypo-methylation (*FAM41C* – gene body; *FLJ39609* – TSS200; *PLEKHN1* – TSS200; *WASH5P* – gene body). The C1orf170 gene body was hyper-methylated in Her2 & LumA tumors and hypo-methylated in Basal-like & LumB tumors. The C1orf170 TSS1500 was hyper-methylated in Her2 tumors and hypo-methylated in Basal-like, LumA, & LumB tumors. The HES4 TSS1500 was hyper-methylated in Basal-like & LumA tumors and hypo-methylated in Her2 & LumB tumors. The ISG15 gene body was hyper-methylated in LumA tumors and hypo-methylated in Basal-like, Her2, & LumB tumors. The NOC2L gene body was hyper-methylated in Her2, LumA, & LumB tumors and hypo-methylated in Basal-like tumors (Table 3 and Supplementary Table S3).

**Table 3:** Differential methylation of the nineteen DMGRs identified

*DMGR*	Basal-like	Her2	Luminal A	Luminal B
AGRN Body	+	+	+	+
C1orf170 Body	−	+	−+	−
C1orf170 TSS1500	−	+	−	−
FAM41C Body	−	−	−	−
FAM41C TSS1500	+	+	+	+
FLJ39609 TSS200	−	−	−	−
HES4 TSS1500	− +	−	+	−
ISG15 Body	−	−	+	−
KLHL17 3′UTR	−+	+	+	−+
KLHL17 Body	−	+	+	+
NOC2L Body	−	+	+	+
PLEKHN1 3′UTR	+	+	+	+
PLEKHN1 Body	+	+	+	+
PLEKHN1 TSS1500	−+	+	+	+
PLEKHN1 TSS200	−	−	−	−
SAMD11 5′UTR	+	+	+	+
SAMD11 Body	+	+	+	+
SAMD11 TSS1500	+	+	+	+
WASH5P Body	−	−	−	−

(−+) DMGRs with both hypo- and hyper-methylated CpGs.

(−) Hypo-methylated.

(+) Hyper-methylated.

**Figure 2:**
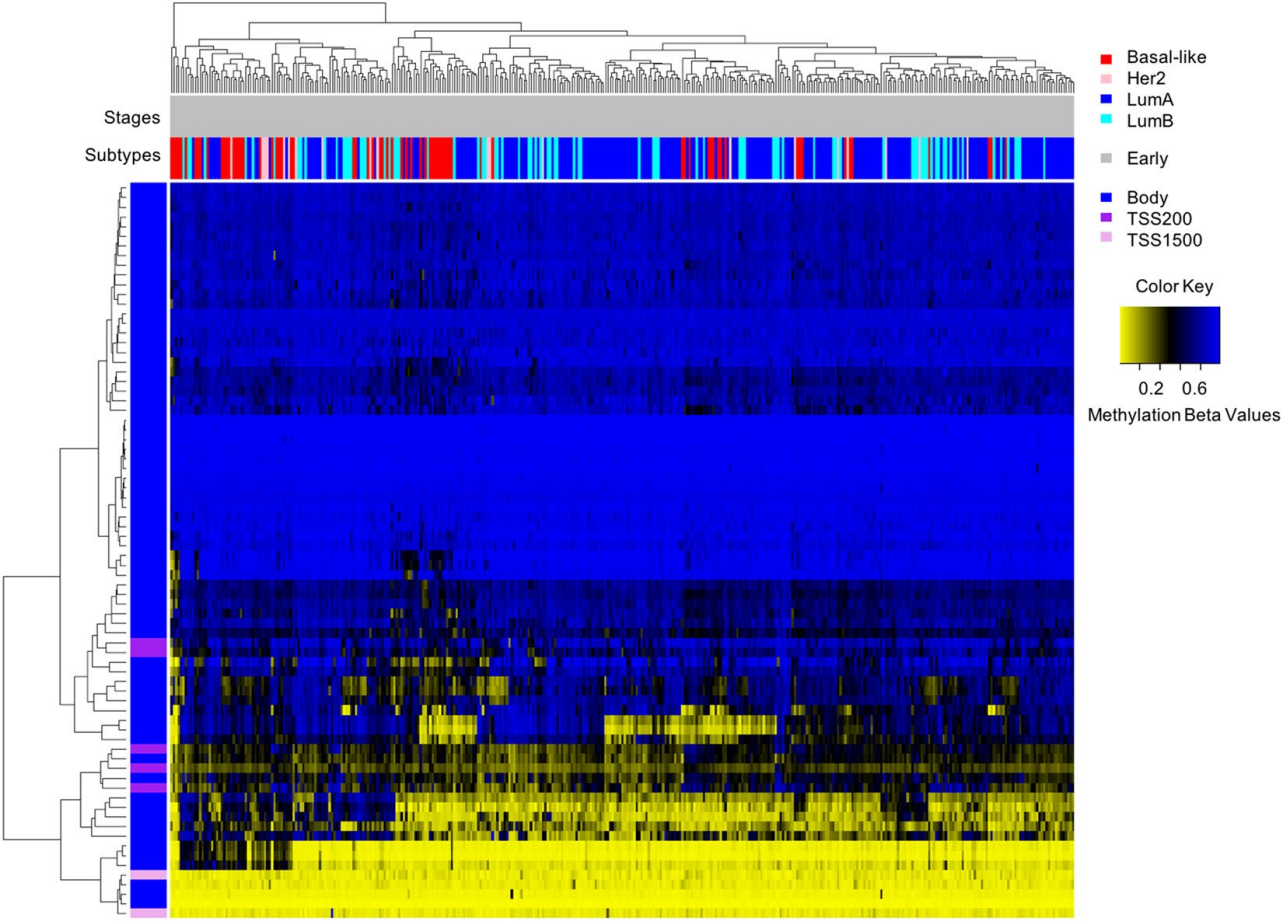
Raw beta value (unadjusted for cellular composition) heatmap of the significantly differentially methylated CpG sites (n = 387) mapping to the common early stage DMGRs (n = 19). The genomic context is given in the vertical color bar and the PAM50 subtype and tumor information (stage and subtype) are given in the horizontal bars. Yellow indicates low methylation and blue indicates high methylation beta values.

All nineteen DMGRs were also identified as differentially methylated in at least one late stage tumor subtype, and thirteen of the nineteen DMGRs were identified as significantly differentially methylated across all tumor subtypes in late stage tumors (Table 2 and Supplementary Table S4). A heatmap of the unadjusted beta values for individual CpGs from the nineteen DMGRs demonstrated grouping of most of the Basal-like tumors separate from a group of mixed Luminal and Her2 tumors (Fig. 2).

### DMGRs on chromosome 1p36

Of the nineteen DMGRs identified, all of them are in eleven genes located on the *p36.3* cytoband of chromosome 1 (Supplementary Figure S4). Chromosome 1p36.3 is the start section of chromosome 1 and of the eleven genes identified, one (*WASH5P*) is located near the very start of the chromosome (chr1:14,362–29,370) and the other ten genes are located end-to-end between chr1:868,071–1,056,116 (Supplementary Figure S4).

Most of the DMGRs tracked to gene body regions: *AGRN, C1orf170, FAM41C, ISG15, KLHL17, NOC2L, PLEKHN1, SAMD11*, and *WASH5P* all had gene body methylation differences. Gene body regions were enriched among early stage tumor DMGRs compared to all other regions: TSS1500, TSS200, 5′ UTR, and 3′ UTR (Fisher’s Exact Test OR = 4.15, 95% CI = 1.04–23.83, *P* = 0.04). All differentially methylated CpG probe IDs are given in Supplementary Table S5. DAVID pathway analysis applied to the top 400 most aberrantly methylated genes in common to the four PAM50 subtypes identified the GO term for the regulation of hormone levels to be significantly enriched (GO:0010817, *FDR* = 0.035, Supplementary Table S6).

### Breast cancer copy number alterations in 1p36

Among these 523 tumors, the prevalence of 1p36.3 copy number alterations (CNAs) was only 1.2% (n = 6), as assessed using the cBioPortal. All observed CNAs were amplifications that affected ten of the eleven genes most distal to the chromosome end. Among the six tumors with 1p36.3 amplification, three were Basal-like, two were Her2-enriched, and one was Luminal A. Exclusive of tumors with copy number alterations, there was one tumor (Her2-enriched), with a truncating mutation in *KLHL17*, and one tumor with a missense mutation in *PLEKHN1* (Basal-like).

### DMGRs impact gene expression

We identified CpG sites with significant correlation of methylation with gene expression for five genes (*AGRN, PLEKHN1*, *KLHL17, SAMD11*, and *FAM41C*), associated with eight DMGRs (Supplementary Table S7 and Supplementary Figures S5–9).

### Validating DMGR hits in an independent dataset

We validated our findings in an independent 450 K methylation data set from 186 tumors and 46 normal tissues described in Fleischer *et al*. (GSE60185). Seventeen of nineteen DMGRs were significantly differentially methylated between tumor and normal tissues in the replication set (all DMGRs at *Q* < 0.01; Table 2), and CpGs in these DMGRs had similar patterns of methylation to those DMGRs identified in the analysis of early stage tumors (Supplemental Figure S10). The remaining two gene regions were also highly ranked in the *q* value distribution (*WASH5P* body: *Q* = 0.07; *ISG15* Body: *Q* = 0.10).

### Reproducibility

All TCGA and validation data is publicly available. We also provide software under an open source license for analysis reproducibility and to build upon our work at https://github.com/Christensen-Lab-Dartmouth/brca_lowstage_DMGRs. The analysis and data files are versioned on Zenodo^22^.

## Discussion

We were interested in identifying common biology underlying breast cancer independent of molecular subtype and cell-type proportion. After applying a reference-free deconvolution algorithm, we observed that early stage tumors harbor differentially methylated gene regions localized entirely to a small region on 1p36.3 shared across four major subtypes. Although DNA methylation alterations are widespread in early stage tumors and prior work has demonstrated alterations that differ among breast tumor subtypes^9, 23^ we observed only 19 DMGRs that overlapped molecular subtypes. All DMGRs tracking to the same region on 1p36.3 suggests that altered regulation of this region contributes to breast carcinogenesis irrespective of disease subtype. An early 2017 study by Lu *et al*.^24^ suggested that hyper-methylation of the gene *RUNX3*, located on 1p36, as an early biomarker and potential therapeutic target in breast cancer.

Previous studies have also identified 1p36.3 as a relevant region to several cancers, with the tumor suppressor homologue of *p53*, the gene *p73*, located on 1p36.3. Corn *et al*.^25^ demonstrated evidence that *p73* is transcriptionally silenced via 5′ CpG island methylation. While we did not identify *p73* as a DMGR in our analysis, the methylation-related association of a tumor suppressor gene with the 1p36.3 region provides strong support of its cancer relevance.

Previously, alterations on chromosome 1 have been observed in breast cancer cell lines and tumors^26^. Additionally, copy number deletions in this region have been shown to be an important precursor in ductal carcinoma *in situ* (DCIS) tumors^27^ and in follicular lymphomas^28^. However, the most prevalent copy number alterations on chromosome 1 are gains on the *q* arm and losses on the *p* arm that do not typically fully encompass our implicated genes on 1p36.3^26, 29–31^. Importantly, the region has also been previously identified to harbor associations between copy number alterations and differential DNA methylation^2^. However, this study was a global analysis aimed to find all copy number breakpoint and methylation associations in a smaller set of breast cancer samples and was not adjusted for cell-type confounding. Conversely, our study was focused on identifying early events in common to breast cancer subtypes and was adjusted for cell-type. Combining this evidence with our study supports a model in which 1p36.3 methylation and copy number alterations are early events in breast carcinogenesis that are not specific to disease subtype. The region is also well-studied and significantly altered in neuroblastoma – the most common solid tissue tumor of childhood^32–36^. A study of meningioma showed that there was no 1p associated loss of heterozygosity (LOH) in grade I tumors, but more than 80% of grade II and III tumors demonstrated LOH^3^. Our analysis focused on early stage breast cancer, where we did not observe any copy number alterations, but it’s possible that the DMGRs we identified indicate increased risk of 1p loss at chromosomal breakpoints, resulting in LOH in late stage tumors.

The biological underpinnings of this region remain elusive^20, 37^ but a systematic understanding of how these specific DMGRs may impact early cancer development may be important for other cancer types and not just breast cancer.

Of the nineteen DMGRs identified, eighteen of them replicated in either one or both late stage tumors and independent validation set analyses. The one DMGR that did not replicate was the *WASH5P body*. This region is located more than 830,000 base pairs (bp) away from the much tighter region spanned by the remaining eighteen DMGRs (~188,000 bp), suggesting a loose association between *WASH5P* and the other ten genes.

There is also additional evidence implicating the potential importance of the identified genes assigned to the differentially methylated regions. For example, in a study of mutational profiles in metastatic breast cancers, *AGRN* was more frequently mutated in metastatic cancers compared with early breast cancers^38^. Similarly, expression of the *HES4* Notch gene is known to be significantly correlated with the presence of activating mutations in multiple breast cancer cell lines, and is associated with poor patient outcomes^39^. In addition, *ISG15* has been implicated as a key player in breast carcinogenesis^40^, though there is conflicting evidence suggesting *ISG15* is both associated with and protective against cancer development^41^. However, the conflicting evidence to date may be related to our observation of *ISG15* hypomethylation in Basal-Like, Her2, and LumB tumors, and hypermethylation in LumA tumors (Supplementary Table S3). Opposing methylation states among tumor subtypes relative to normal tissue may contribute to subtype-specific roles of *ISG15* dysregulation in breast carcinogenesis. Additionally, the *NOC2L* gene has been identified as a member of a group of prognostic genes derived from an integrated microarray of breast cancer studies^42^. We also identified three DMGRs – TSS1500, Body, & 5′UTR – in the *SAMD11* gene, which has significantly reduced expression in breast cancer cells compared to normal tissues^43^, consistent with our findings of *SAMD11* hypermethylation across all four breast cancer subtypes. As DNAm changes were observed consistently and robustly across subtypes, it is likely that several of the other identified genes are cancer initiation factors that require additional study.

Importantly, we validated the identified DMGRs in an independent set of invasive breast tumors and normal tissues. Our validation is strengthened by the lack of molecular subtype assignments in the validation set. The validation of DMGRs in a setting agnostic to intrinsic subtype indicates that differential magnitude or direction of methylation alterations that may be present in different subtypes did not limit our ability to identify significant alterations. A limitation of the validation set is a lack of gene expression data to further investigate relationships between expression and methylation for each gene region. Nevertheless, additional targeted studies on this set of validated genes and gene regions can enhance the understanding of methylation alterations at these DMGRs in breast carcinogenesis.

Caution should be exercised in interpreting the results of the adjusted beta coefficients from the reference-free algorithm. It is unclear if specific disease states are a result of aberrant methylation profiles in specific cell types which then cause changes to cell mixtures, or if the disease state is a result of cell-type proportion differences. Additionally, the unsupervised clustering heatmaps plot unadjusted methylation beta values and do not account for cell type adjustment. Lastly, the DMGR analysis drops CpGs that do not track to gene regions, which may reduce detection of non-genic regions related with breast carcinogenesis.

We identified and validated DMGRs in early stage breast tumors across PAM50 subtypes that are located on chromosome 1p36.3. The observed differential methylation suggests that this region may contribute to the initiation or progression to invasive breast cancer. Additional work is needed to investigate the scope of necessary and sufficient alterations to 1p36.3 for transformation and to more clearly understand the implications of 1p36.3 methylation alterations to gene regulation. Further investigation of DNAm changes to 1p36.3 may identify opportunities for early identification of breast cancer or risk assessment. Lastly, the reference-free approach we used could be applied to methylation datasets from other tumor types to identify potential drivers of carcinogenesis common across histologic or intrinsic molecular subtypes.

## Methods

### Data Processing

We accessed breast invasive carcinoma Level 1 Illumina HumanMethylation450 (450 K) DNAm data (n = 870) from the TCGA data access portal and downloaded all sample intensity data (IDAT) files. We processed the IDAT files with the R package *minfi* using the “Funnorm” normalization method on the full dataset^44^. We filtered CpGs with a detection *P*-value > 1.0E-05 in more than 25% of samples, CpGs with high frequency SNP(s) in the probe, probes previously described to be potentially cross-hybridizing, and sex-specific probes^45, 46^. We filtered samples that did not have full covariate data (PAM50 subtype, pathologic stage^12, 47^) and full demographic data (age and sex). All tumor adjacent normal samples were included regardless of missing data (n = 97, Table 1).

From an original set of 485,512 measured CpG sites on the Illumina 450 K array, our filtering steps removed 2,932 probes exceeding the detection *P*-value limit, and 93,801 probes that were SNP-associated, cross-hybridizing, or sex-specific resulting in a final analytic set of 388,779 CpGs. From 870 TCGA breast tumors, we restricted to primary tumors with available PAM50 intrinsic subtype assignments of Basal-like (n = 86), Her2 (n = 31), Luminal A (n = 279), and Luminal B (n = 127), excluding Normal-like tumors due to limited sample size (n = 18). Lastly, we restricted the final total tumor set to only those with stage assignments resulting in a final analytic sample size of n = 523. These tumors were compared against normal-adjacent tissue samples from the TCGA (n = 124).

### Reference-free cell type adjustment modeling

We stratified samples by PAM50 subtype (Basal-like, Luminal A, Luminal B, Her2) and then by tumor stage dichotomizing as early (stage I and II tumors) and late (stage III and IV tumors)^47^, resulting in eight distinct models. To analyze DNAm differences between tumor and normal tissue and to adjust for effects of cellular heterogeneity across samples, we applied the reference-free deconvolution algorithm from the *RefFreeEWAS* R package to each model adjusting for age^16^. The method estimates the number of underlying tissue-specific cell methylation states contributing to methylation heterogeneity through a constrained variant of NMF^48^. Briefly, the method assumes the sample methylome is composed of a linear combination of the constituent methylomes. It decomposes the matrix of sample methylation values ($Y$) into two matrices ($Y=M{{\rm{\Omega }}}^{T}$), where M is an ${m\; x\; K}$ matrix of m CpG-specific methylations states for K cell types and ${\rm{\Omega }}$ is a ${n\; x\; K}$ matrix of subject-specific cell-types. *K* is selected via bootstrapping *K* = 2…10 and choosing the optimal *K* that minimizes the bootstrapped deviance. Analysis models were run testing the association between each CpG site and Tumor/Normal tissue status, controlling for K-1 underlying cell-types, to generate associated *P*-values. K-1 cell-types were used to prevent multi-collinearity in the statistical models and K cell-types was estimated using *RefFreeEWAS* described above. To correct for multiple comparisons, we converted all extracted *P*-values to *Q*-values using the R package *qvalue* ^49^.

### Identifying differentially methylated gene regions

**Figure 3:**
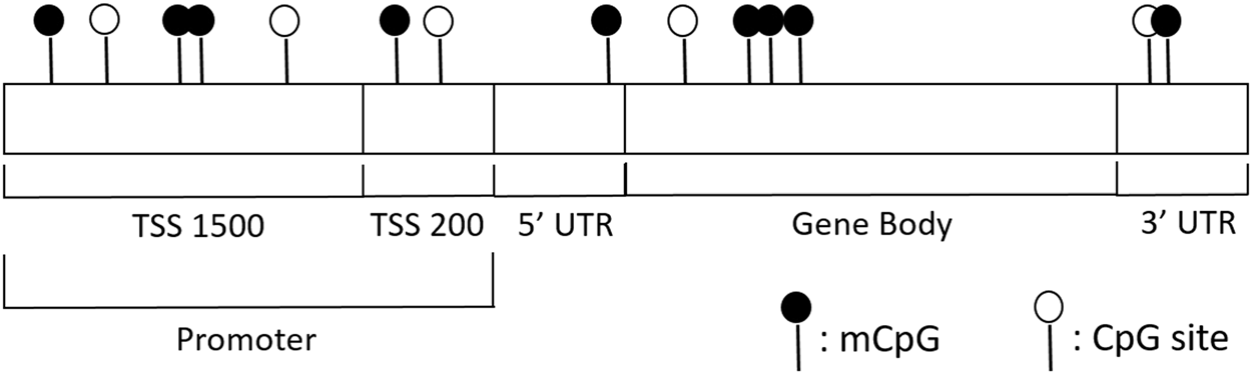
Diagram of CpG sites relative to gene regions (Transcription start sites (TSS1500 & TSS200), Untranslated regions (5′UTR & 3′UTR), and the gene body). Dark circles indicate methylated sites and empty circles indicate unmethylated sites.

To understand the genomic regions with common DNAm alterations we used the grouping of CpGs by gene and region relative to genomic location (transcription start site 1500 (TSS1500, 200–1500 bp upstream of the TSS), TSS200 (0–200 bp upstream of the TSS), 3′ untranslated region (3′UTR), 5′UTR, 1^st^ exon, and gene body). We used this gene-region taxonomy to collapse differentially methylated CpGs, as defined by our *Q*-value cutoff, into specific differentially methylated gene regions (DMGRs). This extended the Illumina 450 K CpG annotation file to allow for a given CpG to be associated with up to two genes depending on the proximity of the CpG site to neighboring genes (Fig. 3).

We defined a differentially methylated CpG as one with a *Q*-value < 0.01 following cell-type adjustment in a specific subtype model compared to normal tissue. To identify DMGR sets for each stage and subtype, we analyzed all eight models independently.

### Pathway Analysis

We performed a DAVID (the database for annotation, visualization and integrated discovery) analysis^50, 51^ for the 400 genes with the lowest median CpG *Q*-values that are in common to all early stage tumors regardless of PAM50 subtype, and extracted enriched Gene Ontology (GO)^52^ and Kyoto Encyclopedia of Genes and Genomes (KEGG)^53^ terms. We selected the top 400 genes based on recommended gene list sizes^51^.

### Copy number, gene expression, and genomic location

We downloaded TCGA Breast Invasive Carcinoma CNA data^9^ and normalized RNAseq using cBioPortal^54^. For the DMGRs we identified, we analyzed the prevalence of copy number alterations and mutations in each gene across all samples, stratified by molecular subtype, via visualization in cBioPortal. Similarly, to determine whether these DMGRs affect gene expression of their target gene, we calculated Spearman correlations of DNAm beta values in significant CpGs (*Q* < 0.01) to matched sample Illumina HiSeq gene expression data. We used a Bonferroni correction to determine significant expression differences, resulting in an acceptance alpha value of 9.36E-5.

### Validation

To confirm the identified early stage DMGRs in common among intrinsic molecular subtypes we applied the analysis workflow to TCGA late stage tumors and an independent validation set (GSE60185)^21^. The validation set includes samples of ductal carcinoma *in situ* (DCIS), mixed, invasive, and normal histology collected from Akershus University Hospital and from the Norwegian Radium Hospital. We analyzed only the invasive samples compared to normal samples using the same bioinformatics pipeline of quality control CpG filtering steps and normalization procedures. However, we did not have complete age information or intrinsic subtype assignments for the validation set and the models are not adjusted for age or stratified by subtype. This resulted in a single model comparing 186 invasive tumors with 46 normal controls measured across 390,253 CpGs.

## Electronic supplementary material

Supplemental information
