# Supplementary material for: Deconvolution of DNA methylation identifies differentially methylated gene regions on 1p36 across breast cancer subtypes
Source: Sci Rep. 2017 Sep 14;7:11594. doi: 10.1038/s41598-017-10199-z (PMC5599639; doi:10.1038/s41598-017-10199-z)
Supplement: Supplementary file 1 — Supplemental information [file 41598_2017_10199_MOESM1_ESM.pdf]

Deconvolution of DNA methylation identifies differentially methylated gene regions on 1p36 across breast cancer subtypes

Alexander J. Titus[1,a,d], Gregory P. Way[1,b], Kevin C. Johnson[c,d], and Brock C. Christensen*[d,e,f]

[1]Authors contributed equally
[a] Program in Quantitative Biomedical Sciences, Geisel School of Medicine at Dartmouth, Hanover, NH 03755
[b] Genomics and Computational Biology Graduate Program, University of Pennsylvania, Philadelphia, PA 19104
[c] The Jackson Laboratory for Genomic Medicine, Farmington, CT 06032, USA
[d]Department of Epidemiology,
[e] Department of Molecular and Systems Biology
[f] Department of Community and Family Medicine, Geisel School of Medicine at Dartmouth, Hanover, NH 03755

CORRESPONDING AUTHOR:
* 1 Medical Center Drive, Williamson Level 6, HB7650, Lebanon, NH 03766; Phone: 603-650-1828; Fax: 603-650-1840 Brock.Christensen@Dartmouth.edu

AUTHOR EMAILS:
AT: Alexander.J.Titus.gr@dartmouth.edu
GW: GregWay@upenn.edu
KJ: Kevin.C.Johnson@jax.org
BC: Brock.Christensen@dartmouth.edu

SUPPLEMENTAL FIGURES:

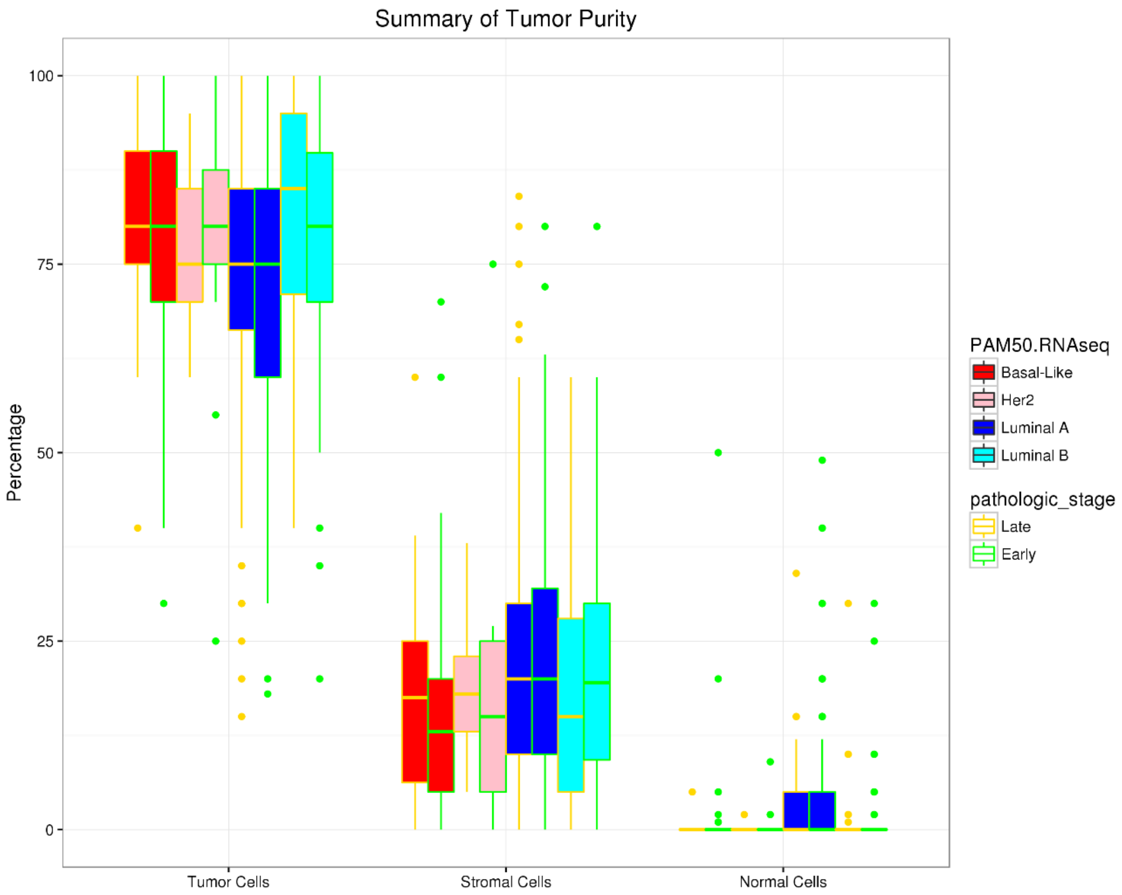

Supplementary Figure S1. *Box plots show the distribution of tumor purity across all subtypes for both early and late stages of the TCGA dataset. The measurements estimated by TCGA are based on histology slides and indicate the estimated distribution of the number of tumor cells, stromal cells, and normal cells in each sample. See the NCI CDE Browser for more details.*

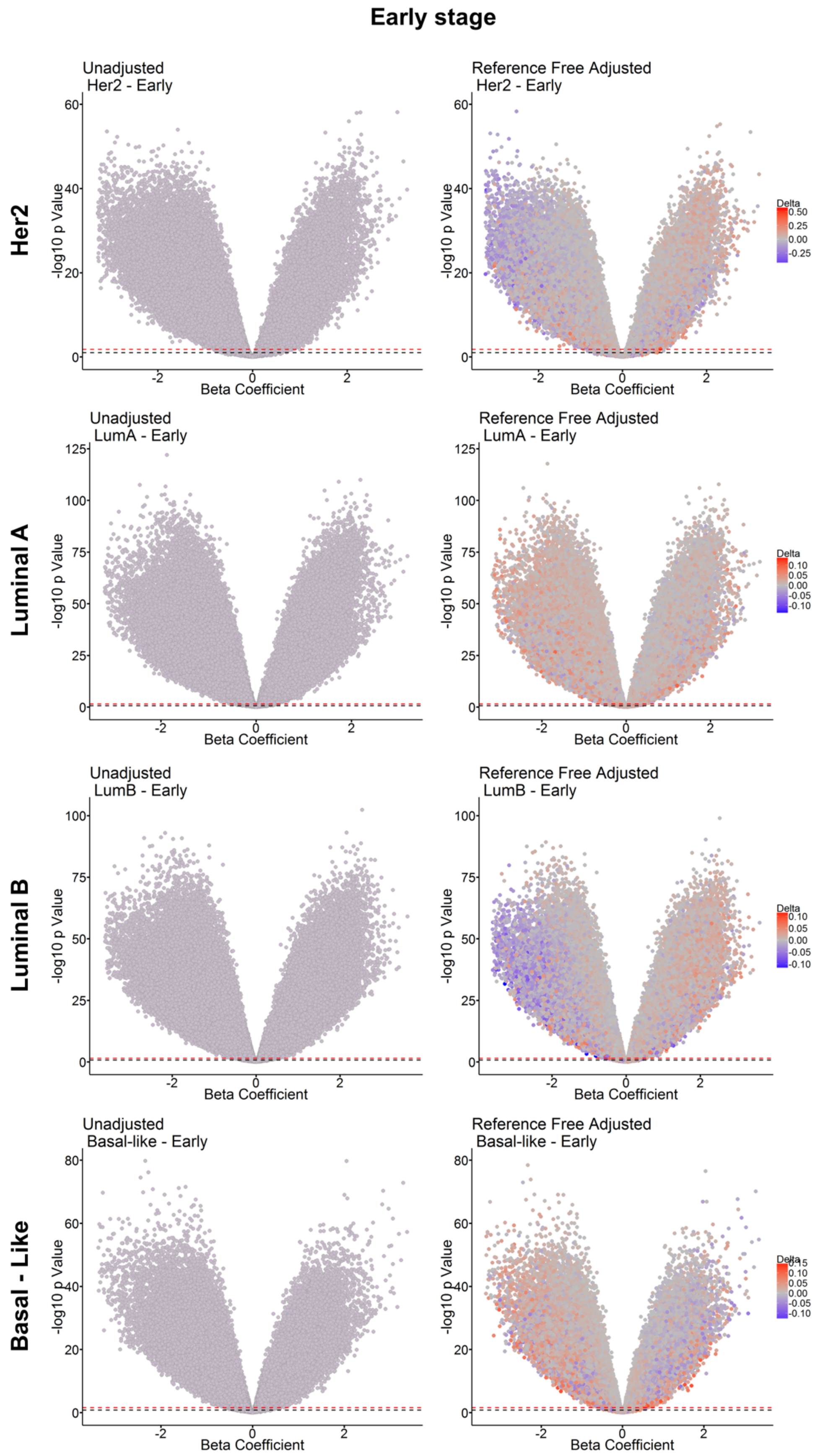
Early stage
Her2
Luminal A
Luminal B
Basal - Like
Unadjusted
Her2 - Early
Reference Free Adjusted
Her2 - Early
Unadjusted
LumA - Early
Reference Free Adjusted
LumA - Early
Unadjusted
LumB - Early
Reference Free Adjusted
LumB - Early
Unadjusted
Basal-like - Early
Reference Free Adjusted
Basal-like - Early
-log10 p Value
Beta Coefficient
Delta

Supplementary Figure S2. *Volcano plots from the early-stage models. The left most panel in each model indicates unadjusted P values and the right panel indicates RefFree adjusted P values. Each point represents a CpG considered in the model and the color of the points represents the change in the beta coefficient following adjustment (delta value). The red lines indicate a Q value cutoff of 0.01 and the black lines indicate a Q value cutoff of 0.05.*

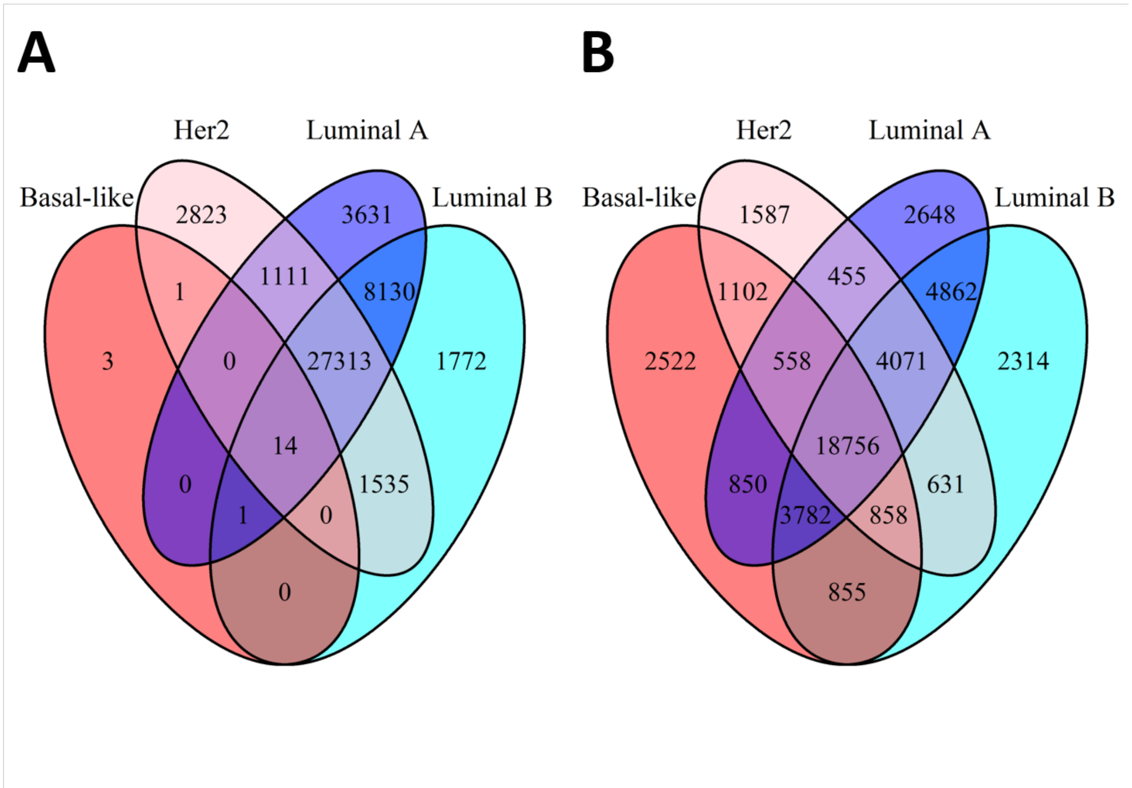

Supplementary Figure S3. *Venn diagram depicting overlapping Illumina annotation file UCSC regions between (A) early and (B) late stage tumors stratified by subtype. The regions consist of mappings relative to CpG island definitions (e.g. <Gene Name> N_Shore).*

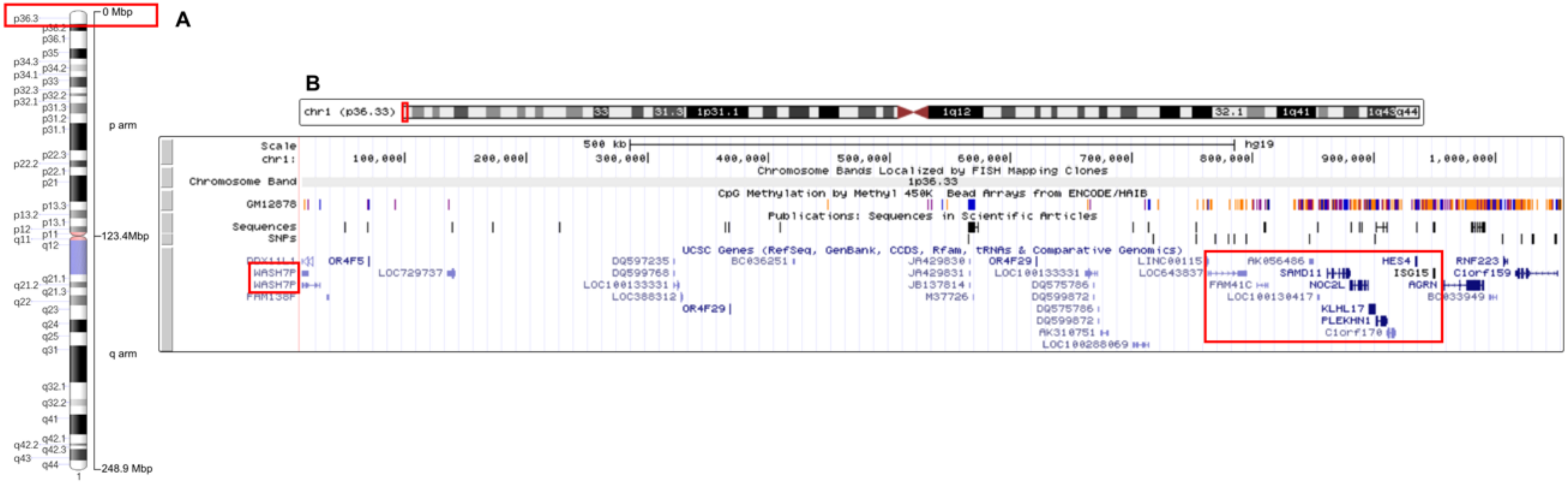

Supplementary Figure S4. *Diagram of chromosome 1. (A) The entire chromosome 1 with regions annotated. (B) A zoomed in view of chromosome 1p36.3 with each identified gene annotated on a track and highlighted in red boxes indicating a gene cluster between base pairs 868,071 - 1,056,116.*

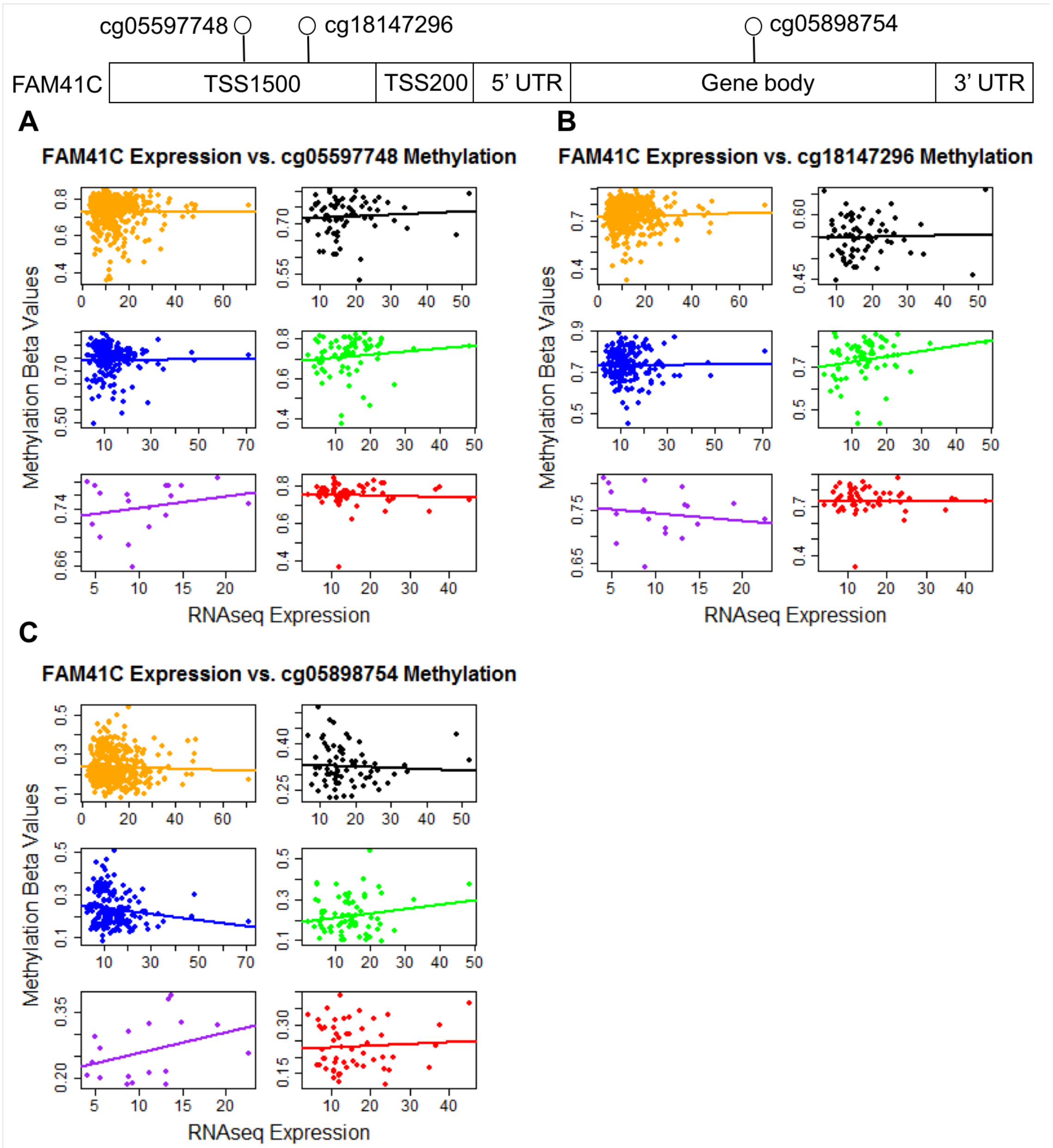

Supplementary Figure S5. *The relationship between differentially methylated CpG sites and FAM41C gene expression in early stage tumors and normal tissue with matched RNAseq samples stratified by PAM50 subtype. All tumors (orange), all normal tissue (black), Luminal A (blue), Luminal B (green), Her2 (purple), and Basal-like (red) are given in the different facets of the figure. Above the figure is a diagram of which DMGR the CpG sites are located within.*

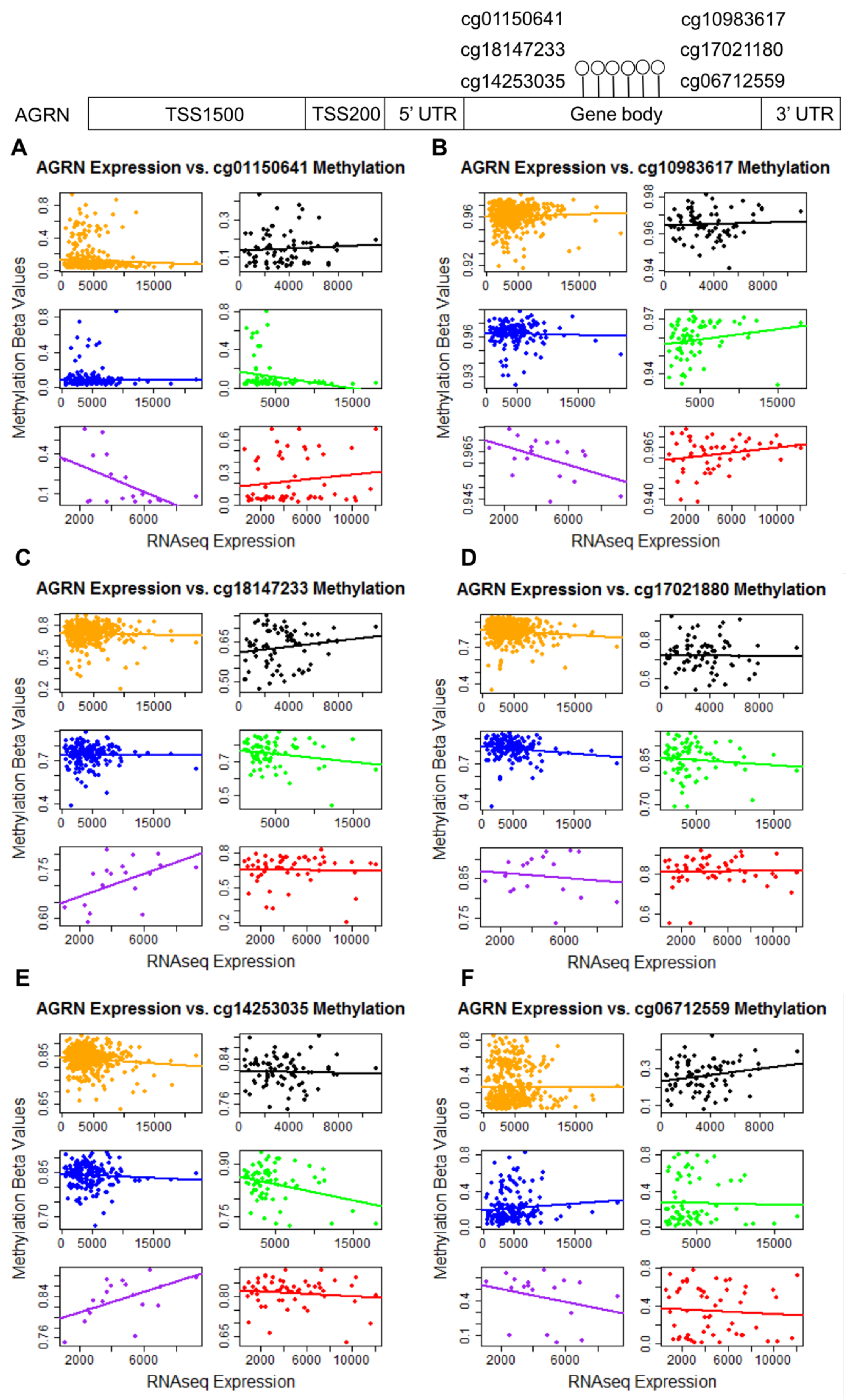
cg01150641
cg18147233
cg14253035
cg10983617
cg17021180
cg06712559
AGRN
TSS1500
TSS200
5' UTR
Gene body
3' UTR
A
AGRN Expression vs. cg01150641 Methylation
B
AGRN Expression vs. cg10983617 Methylation
C
AGRN Expression vs. cg18147233 Methylation
D
AGRN Expression vs. cg17021880 Methylation
E
AGRN Expression vs. cg14253035 Methylation
F
AGRN Expression vs. cg06712559 Methylation
Methylation Beta Values
RNAseq Expression

Supplementary Figure S6. *The relationship between differentially methylated CpG sites and AGRN gene expression in early stage tumors and normal tissue with matched RNAseq samples stratified by PAM50 subtype. All tumors (orange), all normal tissue (black), Luminal A (blue), Luminal B (green), Her2 (purple), and Basal-like (red) are given in the different facets of the figure. Above the figure is a diagram of which DMGR the CpG sites are located within.*

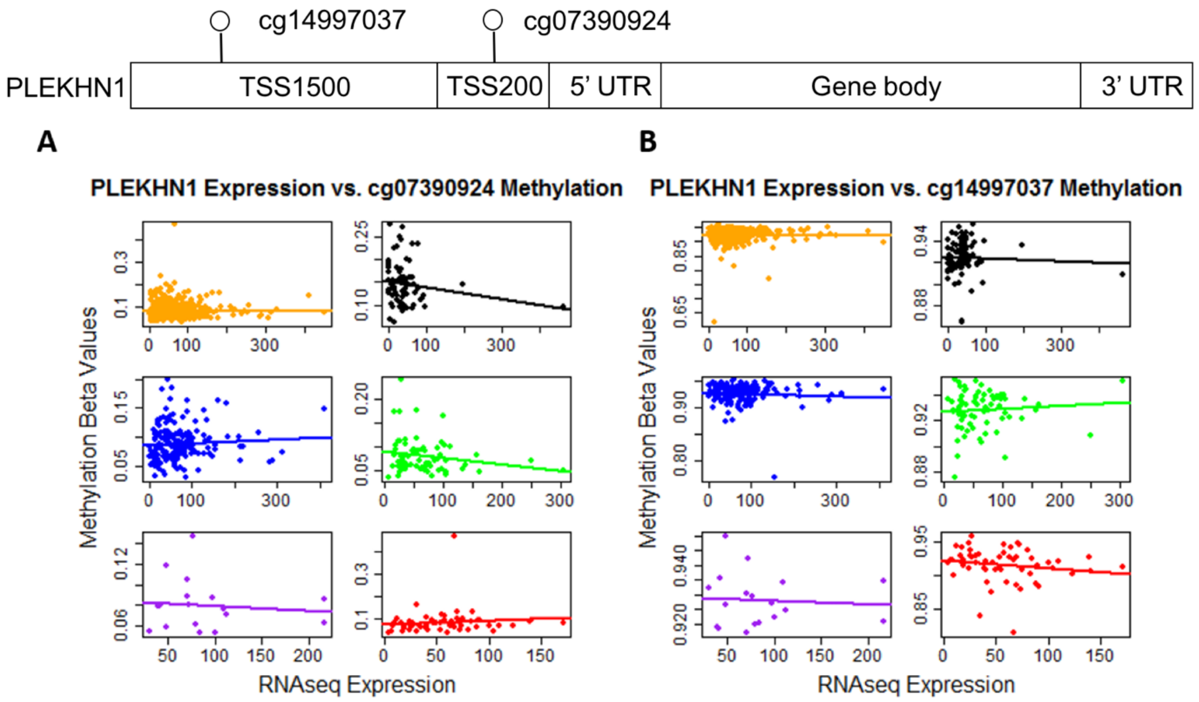

Supplementary Figure S7. *The relationship between differentially methylated CpG sites and PLEKHN1 gene expression in early stage tumors and normal tissue with matched RNAseq samples stratified by PAM50 subtype. All tumors (orange), all normal tissue (black), Luminal A (blue), Luminal B (green), Her2 (purple), and Basal-like (red) are given in the different facets of the figure. Above the figure is a diagram of which DMGR the CpG sites are located within.*

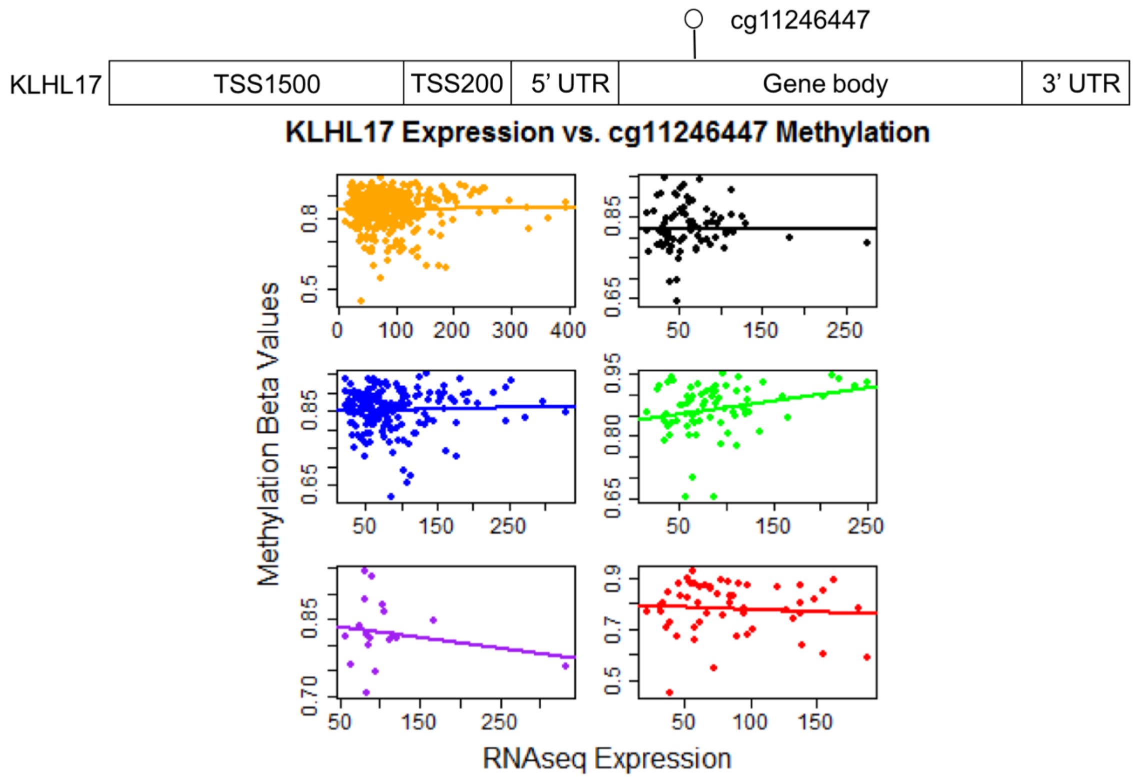

Supplementary Figure S8. *The relationship between differentially methylated CpG sites and KLHL17 gene expression in early stage tumors and normal tissue with matched RNAseq samples stratified by PAM50 subtype. All tumors (orange), all normal tissue (black), Luminal A (blue), Luminal B (green), Her2 (purple), and Basal-like (red) are given in the different facets of the figure. Above the figure is a diagram of which DMGR the CpG sites are located within.*

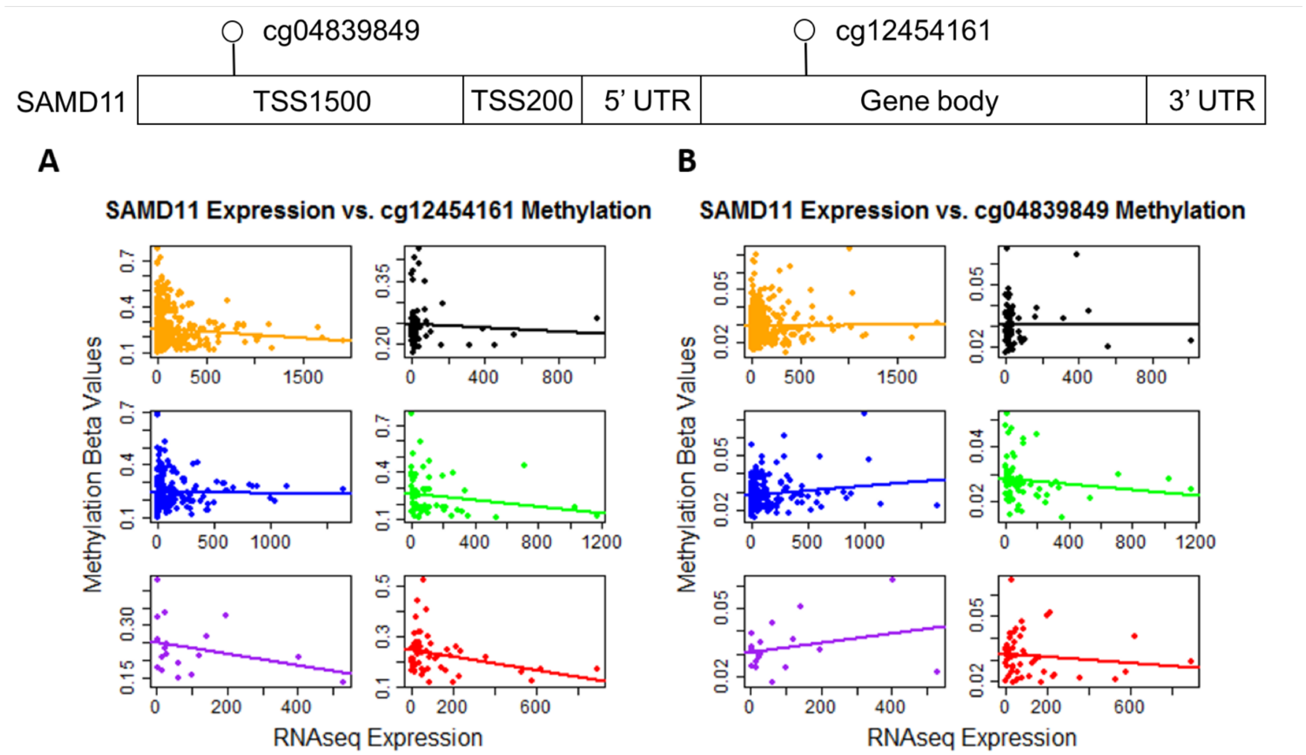

Supplementary Figure S9. *The relationship between differentially methylated CpG sites and SAMD11 gene expression in early stage tumors and normal tissue with matched RNAseq samples stratified by PAM50 subtype. All tumors (orange), all normal tissue (black), Luminal A (blue), Luminal B (green), Her2 (purple), and Basal-like (red) are given in the different facets of the figure. Above the figure is a diagram of which DMGR the CpG sites are located within.*

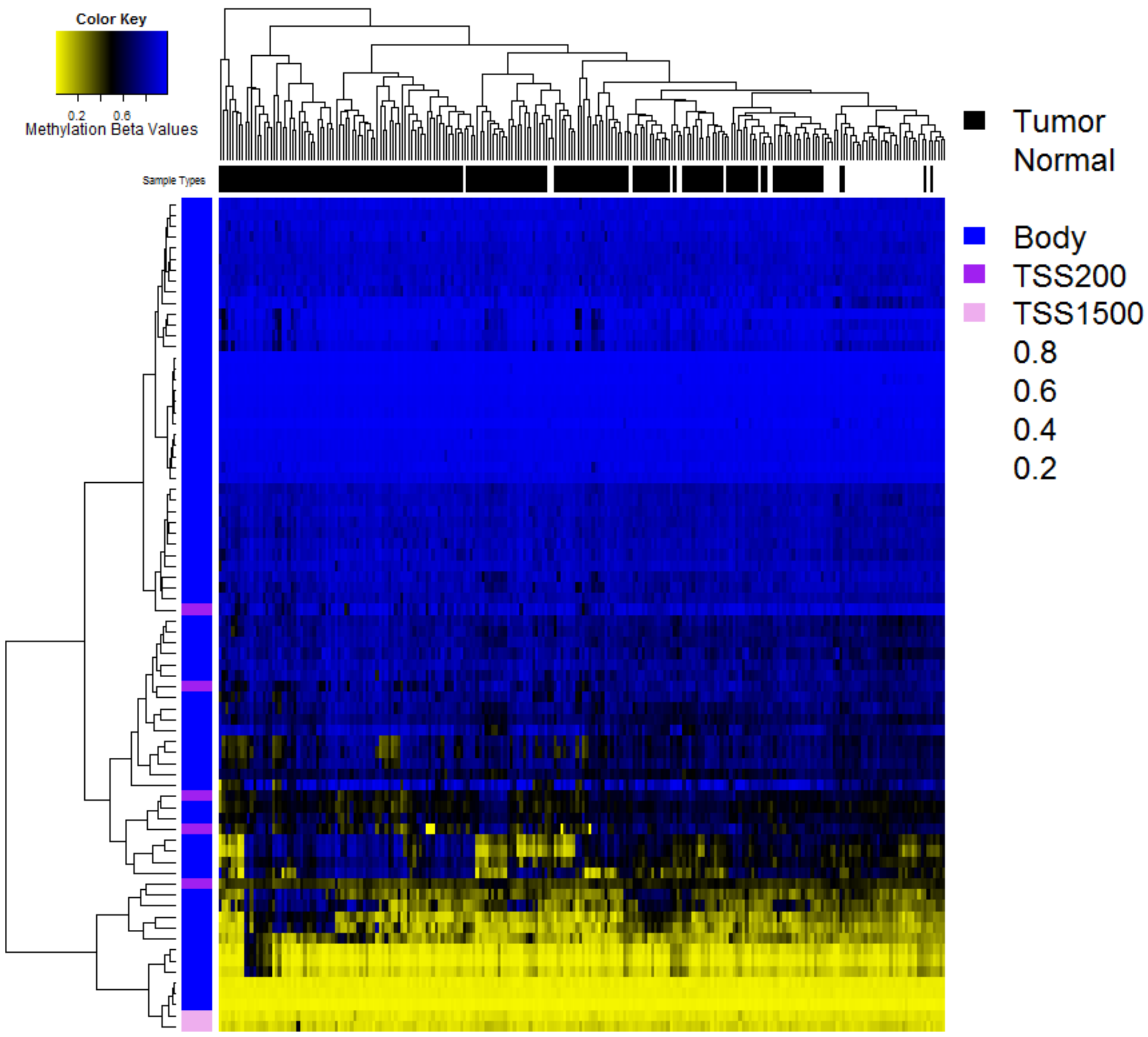

Supplementary Figure S10. *Results from the validation set (Fleischer et al 2014; GSE60185). Validation set raw (unadjusted) beta value heatmap of the significantly differentially methylated CpG sites in the common early stage differentially methylated gene regions (DMGRs) identified in the initial analysis. The genomic context is given in the vertical color bar (blue = gene body, dark pink = TSS200, light pink = TSS1500) and tumor vs. normal status is given in the horizontal color bar (black = tumor, white = normal tissue). In the heatmap, yellow indicates low methylation and blue indicates high methylation.*

SUPPLEMENTAL TABLES:

Due to size limitations of this document and the size of the supplemental tables available for this manuscript, supplemental tables may be found at the following DOI link:

DOI: 10.5281/zenodo.400247
